# Macro-structural predictors of Australian family day care quality

**DOI:** 10.3389/fpubh.2023.1114256

**Published:** 2023-05-18

**Authors:** Vincent Char, Linda J. Harrison, Hui Li

**Affiliations:** ^1^Macquarie School of Education, Macquarie University, Sydney, NSW, Australia; ^2^Shanghai Institute of Early Childhood Education, Shanghai Normal University, Shanghai, China

**Keywords:** family day care, national quality framework, systemic features, early childcare, QRIS

## Abstract

**Introduction:**

This study explores the predictive power of macro-structural characteristics on quality rating and improvement system (QRIS) outcomes of Family Day Care (FDC) services in Australia.

**Methods:**

The dataset consisted of 441 FDC National Quality Standard (NQS) ratings from all Australian states and territories, with overall ratings of Exceeding NQS, Meeting NQS, Working Towards NQS, or Significant Improvement Required.

**Results:**

Multinomial logistic regressions confirmed that management type, community socioeconomic status (SES), level of urbanization, and government jurisdiction explained 6.9 to 19.3% of the variation in QRIS outcomes. Results indicated that lower FDC NQS ratings were more likely for (1) private for-profit vs. not-for-profit; (2) low-SES vs. high-SES area; and (3) regional or remote area vs. metropolitan. State/territory jurisdiction also influenced NQS ratings.

**Discussion:**

These findings imply the need for policy attention to inequalities in FDC quality associated with systemic and organizational differences. Greater effort is needed to promote equality and equity in FDC services.

## Introduction

Early childhood education and care (ECEC) attendance can positively affect young children’s early learning and development, academic success, and socioeconomic mobility (1). Family Day Care (FDC), also known as family child care, is a globally accessed form of ECEC. FDC is family-based, allowing educators to care for children in their own homes, and children to attend with siblings, including those attending primary school (2). FDC educators care for over 125,000 children in Australia (3). Yet, FDC has consistently received lower ratings than other types of education and care on an Australian quality rating and improvement system (QRIS), the National Quality Standard (NQS) Assessment and Rating system [Australian Children’s Education & Care Quality Authority (4)].

Multiple layers of structure, deemed “macro-structural” variables by Harrison et al. (5), may influence ECEC quality (5, 6). The layers of structure that predict ECEC quality have been identified as systemic, organizational, classroom, and staff (7). Unfortunately, few studies have examined how macro-structural features might predict quality childcare provision. To fill this gap, this research aims to explore the predictive power of three systemic characteristics (community socioeconomic status (SES), level of urbanization, government jurisdiction) and one organizational characteristic (management type) on QRIS outcomes in a national sample of Australian FDC services.

### Australian family day care and the national quality standard

Australian FDC educators are self-employed providers of regulated home-based ECEC and are registered with and supported by the coordination units of approved FDC schemes (8). Educators can care for up to seven children, including their own, with a maximum of four children under school age, in their FDC residence or approved venue. FDC schemes employ coordinators and fieldworkers to provide operational support and monitor the uptake of regulations and quality assurance standards (9, 10). Currently, Australia’s 500 FDC schemes make up 4% of education and care services (3, 11).

Education and care services have been assessed against the NQS since 2012. The NQS sets a national quality benchmark for publicly funded education and care services in Australia. It is part of the National Quality Framework (NQF), a unified national system for education and care introduced in 2008 (12). Service quality is assessed against seven quality areas (QAs), each underpinned by a set of standards and elements, and an overall NQS rating. Of particular importance to educator stakeholders are QA 1 (educational program and practice) and QA 5 (relationships with children). QA 1 emphasizes curricula process quality while QA 5 emphasizes building responsive and respectful relationships with children (4). The role of family and community stakeholders are reflected through QA6 (collaborative partnerships with families and communities). QA 6 emphasizes active communication, consultation and collaboration between FDC schemes with families and communities. The NQS comprises four ratings: (1) Significant Improvement Required (SIR); (2) Working Towards NQS (WT); (3) Meeting NQS (MEET); and (4) Exceeding NQS (EXCEED). A rating of “Excellent” is available upon application to services that score EXCEED in all seven QAs (4).

### Macro-structural characteristics

The importance of ECEC quality has led to a plethora of research into factors that predict quality (e.g. (13, 14),). Structural indicators (educator-to-child ratios; group size; educator qualifications) are the primary drivers of process quality (7, 15). In Australia, consistency of educator-to-child ratios, group size, and educator qualifications is ensured across all eight states and territory jurisdictions by the NQF (16).

#### Management type

For-profit ECEC services are generally reported to be lower quality than not-for- profit services (5, 17–20). Cleveland and Krashinsky (19) theorized that the lower quality of care for for-profit services was due to thick and thin markets, resulting from high and low levels of middle- and upper-income families in the local area, respectively. Thick markets have been theorized to support investment in higher quality from not-for-profit service providers through choices relating to lower child–staff ratios, better-educated staff and directors, higher fees and higher rates of professional development for staff (19).

#### Community socioeconomic status

Low SES levels have been theorized to be correlated with thin markets, resulting in lower ECEC quality (19). Significantly less availability of ECEC in low SES areas and a lower average quality of care have been reported in low SES neighborhoods than in more advantaged neighborhoods (21). Lower proportions of children receiving public childcare subsidies has been associated with higher global quality in a study of FDC services in four American States (22). In Australia, demand for ECEC services was more likely to outpace local supply in low SES areas as compared to high SES areas (23). Community SES was found to indirectly influence access to ECEC quality (24).

#### Level of urbanization

Research has indicated that rural areas offer lower-quality education and care services than urban areas (25, 26). For example, a shortage of qualified teachers affects ECEC service quality in rural Vietnam (27). In addition, kindergartens in rural areas of Zhejiang province in China had problems recruiting high-quality teachers and funding the purchase of furnishings, equipment, and educational materials (28). In Australia, NQS assessment and rating records indicate that ECEC services in metropolitan regions receive higher ratings than services in regional or remote areas of Australia (4). Similarly, ECEC services supply is generally higher in metropolitan regions than that in regional and remote Australia (23). There was a complete absence of ECEC supply in many regional towns in Australia, for instance, about 360 towns with a population under 1,500 did not have a center-based day care (23). This demand–supply gap of ECEC in Australia may be linked to the quality differences.

#### Government jurisdiction

Regulatory differences between state government jurisdictions have influenced ECEC quality in the United States of America (22, 29). For example, a study of 120 FDC services in Kansas, Nebraska, Missouri, and Iowa linked different regulatory requirements with different FDC quality levels (22). The state the ECEC service was located in was a significant predictor of observed quality as measured by total scores on the Family Day Care Rating Scale (30) and the subscale scores for Tone and Discipline and Provisions for Learning and Health (29). Within Australia, NQS records and research into center-based ECEC services have revealed varying levels of quality and quality improvement for different states and territories (4, 5).

### Study aims

This study aimed to examine the influences of these four macro-structural characteristics on differences in FDC quality as assessed through Australia’s NQS A&R system. An overall rating of “SIR” or “WT” indicates lower levels of quality. “MEET” means that all standards have been met, while a rating of “EXCEED” indicates higher levels of quality. An overarching question about whether macro-structural factors predict FDC quality was operationalized into three analysis questions:

Do management type, SES, level of urbanization, and jurisdiction predict the likelihood of an FDC scheme…

1. attaining a rating of “WT”/“SIR” vs. a rating of “MEET”?
2. attaining a rating of “WT”/“SIR” vs. a rating of “EXCEED”?
3. attaining a rating of “MEET” vs. a rating of “EXCEED”?

## Method

### Population

The study used Australia’s national data repository of NQS ratings to undertake secondary data analysis. Data for 441 FDC schemes in Australia that had completed an NQS assessment by December 2020 (11) were analyzed. The ACECQA repository also included management and location information about the FDC scheme.

### Measures

#### Quality ratings

The overall quality of each scheme was assessed following the NQS assessment and rating process as:

1. *Significant Improvement Required*: Service does not meet 1 of the 7 QAs or a section of the legislation, and there is a significant risk to the safety, health, and wellbeing of children. The regulatory authority will take immediate action;
2. *Working Towards NQS*: Service provides a safe education and care program. There are one or more QAs identified for improvement;
3. *Meeting NQS*: Service provides quality education and care in all seven QAs;
4. *Exceeding NQS*: Service goes beyond the requirements of the NQS in at least four of the seven QAs.

Frequency analysis for the assessed 441 schemes showed that only 4 (0.9%) schemes had received an overall NQS rating of “SIR,” 209 (47.4%) schemes received a rating of “WT,” 170 (38.5%) schemes received a rating of “MEET,” and 58 (13.1%) schemes received a rating of “EXCEED.”

### NQS version

Of the 441 FDC schemes, 205 (46.5%) had been assessed under the original 2012 version of the NQS, and 236 (53.5%) had been assessed with the 2018 version.

### Management type

Management type for the FDC schemes was identified by four categories: (1) for-profit; (2) not- for-profit – community-based organizations; (3) not-for-profit – other organizations; and (4) not-for-profit - local government. Frequency analysis showed that for-profit FDC schemes were the largest management type (*n* = 248, 56.2%), followed by local government FDC schemes (*n* = 79, 17.9%), community-based FDC schemes (*n* = 72, 16.3%), and FDC schemes run by other not-for-profit organizations (*n* = 42, 9.5%).

### Community socioeconomic status

Community SES was measured by the Socioeconomic Indexes for Areas (SEIFA) which is a composite of four indexes, including two indexes of advantage, one of education and occupation, and one of economic resources (31). Higher positions on the SEIFA quintiles (level 5) denote more elevated levels of advantage than lower positions (level 1) (31). The majority of FDC schemes were located in communities with SEIFA quintile 1 (*n* = 181, 43.5%), followed by quintile 2 (*n* = 88, 21.2%), quintiles 3 and 4 (*n* = 58, 13.9% each), and quintile 5 (*n* = 31, 7.5%). The remaining 25 FDC schemes were not matched to a SEIFA level.

### Level of urbanization

The level of urbanization was measured by the Accessibility and Remoteness Index of Australia (ARIA+) which represents a general access model covering education, health, shopping, public transport, and financial/postal services (32). ARIA+ levels of urbanization are based on road distance from over 12,000 population localities across Australia to the nearest service center locality based on population size, which is used as a proxy measure of service availability (33). ARIA+ identifies five categories: (1) Major Cities (populations of approximately 250,000 persons or more); (2) Inner Regional cities or towns (populations of approximately 48,000 to 249,999 persons); (3) Outer Regional towns (populations of approximately 18,000 to 47,999 persons); (4) Remote townships (populations of approximately 5,000 to 17,999 persons); (5) Very Remote areas (populations of approximately 1,000 to 4,999 persons). The majority of FDC schemes were located in major cities (*n* = 300, 68.0%), followed by inner regional (*n* = 88, 20.0%), outer regional (*n* = 47, 10.7%), remote (*n* = 4, 0.9%), and very remote (*n* = 2, 0.4%) areas of Australia.

### Jurisdiction

There are eight government jurisdictions in Australia: six states, New South Wales, Victoria, Queensland, Western Australia, South Australia, Tasmania, and two territories, Australia Capital Territory (ACT) and Northern Territory. New South Wales had the most FDC schemes (*n* = 147, 33.3%), followed by Victoria (*n* = 133, 30.2%), Queensland (*n* = 100, 22.7%), Western Australia (*n* = 30, 6.8%), South Australia (*n* = 12, 2.7%), Tasmania (*n* = 10, 2.3%), ACT (*n* = 6, 1.4%), and the Northern Territory (*n* = 3, 0.7%).

### Analysis plan

Cross-tabulations were conducted to assess the distribution of FDC quality ratings for each of the four macro-structural characteristics and the NQS version (2012 vs. 2008). Regression analysis was employed to examine the influences of each macro-structural characteristic on FDC quality. Because the outcome variable was categorical, we chose multinomial logistic regression (MLR), which predicts the probability that an observation falls into one of three or more categories of a dependent variable (DV) based on one or more independent variables (IVs) (34). The IVs can be either continuous or categorical.

For our final analyses, we conducted two sets of MLRs using the 3-category DV. The first tested the likelihood of each macro-structural characteristic predicting a rating of WT/SIR versus a rating of (1) MEET and (2) EXCEED. The second set of MLRs used “MEET” as the reference category to test the predictive effects of each of the four macro-structural characteristics predicting a rating of “MEET” versus (1) “EXCEED” and (2) “WT”/ “SIR.” The validity of the models was tested with a chi-square test and confirmed with Pearson’s goodness of fit test. The nagelkerke *R*^2^ (a pseudo *R*^2^) was used to estimate the variation in overall NQS rating scores explained by the model (35). Finally, Odds Ratios (ORs) were used to determine the nature of the predictive effects for the IVs within each model. The Odds Ratio (OR) computes the chances of a particular event happening in comparison to the event not happening (36). To fully explore the contrasts (ORs) within each IV, MLRs were repeated with different reference categories: three MLRs for management type, four for SES, two for the level of urbanization, and three for jurisdiction.

The version of the NQS (2012/2018) was included as a covariate. This increased the number of statistical tests, with a corresponding increase in the potential for a Type I error (i.e., a false positive). A Type I error risk was accounted for by dividing the standard critical value of 0.05 by two, the total number of predictor variables, to yield a critical value of 0.025 (37).

## Results

### Descriptive analysis

The distribution of FDC schemes across the four NQS quality ratings by each macro-structural characteristic and the NQS version are presented in Table 1. In terms of management type, results indicated that a higher proportion of private for-profit FDC schemes received ratings of “WT” (61.7%) compared to community-based not-for-profit FDC schemes (30.6%), FDC schemes managed by other not-for-profit organizations (26.2%) or FDC schemes local government-provided FDC (29.1%). Conversely, results suggested that private for-profit FDC schemes were less likely to achieve a rating of “MEET” or “EXCEED” (32.3 and 5.2%, respectively) than community-based not-for-profit FDC (50.0 and 18.1%), FDC schemes managed by other not- for-profit organizations (50.0 and 21.4%) or FDC schemes managed by local government, such as city councils (41.8 and 29.1%).

**Table 1:** Distribution of overall NQS quality ratings by macro-structural characteristics and NQS version.

	All FDC schemes	Significant improvement required			Working towards NQS		Meeting NQS		Exceeding NQS	
	*n*	%	*n*	%	*n*	%	*n*	%	*n*	%
Management type										
Private for-profit	248	56.2	2	0.8	153	61.7	80	32.3	13	5.2
Not-for-profit – community-based	72	16.3	1	1.4	22	30.6	36	50.0	13	18.1
Not-for-profit other organizations	42	9.5	1	2.4	11	26.2	21	50.0	9	21.4
Not-for-profit local government	79	17.9	0	0.0	23	29.1	33	41.8	23	29.1
Quintile 1	181	43.5	1	0.6	91	50.3	67	37.0	22	12.2
Quintile 2	88	21.2	1	1.1	44	50.0	33	37.5	10	11.4
Quintile 3	58	13.9	0	0.0	21	36.2	34	58.6	3	5.2
Quintile 4	58	13.9	0	0.0	24	41.4	21	36.2	13	22.4
Quintile 5	31	7.5	1	3.2	11	35.5	11	35.5	8	25.8
Level of Urbanization										
Major cities in	300	68.0	2	0.7	159	53.0	103	34.3	36	12.0
Australia Inner regional	88	20.0	1	1.1	31	35.2	40	45.5	16	18.2
Australia Outer regional	47	10.7	1	2.1	16	34.0	24	51.1	6	12.8
Australia Remote Australia	4	0.9	0	0.0	3	75.0	1	25.0	0	0.0
very remote Australia	2	0.5	0	0.0	0	0	2	100.0	0	0.0
Jurisdiction New South Wales	147	33.3	2	1.4	69	46.9	61	41.5	15	10.2
Victoria	133	30.2	1	0.8	70	52.6	48	36.1	14	10.5
Queensland	100	22.7	0	0.0	35	35.0	46	46.0	19	19.0
Western Australia	30	6.8	0	0.0	22	73.3	3	10.0	5	16.7
South Australia	12	2.7	0	0.0	8	66.7	3	25.0	1	8.3
Tasmania	10	2.3	1	10.0	4	40.0	3	30.0	2	20.0
ACT	6	1.4	0	0.0	1	16.7	4	66.7	1	16.7
NT	3	0.7	0	0.0	0	0	2	66.7	1	33.3
NQS Version										
2012	205	46.5	0	0.0	98	47.8	67	32.7	40	19.5
2018	236	53.5	4	1.7	111	47.0	103	43.6	18	7.6

The pattern of distributions of NQS ratings across the five SEIFA levels suggested an association between quality and community SES. A lower proportion of FDC schemes located in more disadvantaged communities (SEIFA quintiles 1 and 2) were rated as “EXCEED” (12.2 and 11.4%, respectively) compared to FDC schemes located in SEIFA quintiles 4 and 5 (22.4 and 25.8%). However, there was inconsistent evidence of a linear relationship: for example, only 5.2% of FDC schemes located in SEIFA quintile 3 were rated as Exceeding NQS.

Results for the level of urbanization indicated that FDC schemes located in major cities were more likely to be rated as “WT” (53.0%) than FDC schemes in inner regional and outer regional areas (35.2 and 34.0%, respectively). The converse pattern was evident for ratings of “MEET,” which were more evident in regional areas (inner = 45.5%; outer = 51.1%) than in cities (34.3%).

The results for jurisdiction suggested some differences between the states. FDC schemes in Queensland had a lower proportion of “WT” ratings (35.0%) than FDC schemes in New South Wales (46.9%), Victoria (52.6%), Western Australia (73.3%) and South Australia (66.7%), and a higher proportion of “Exceeding NQS” (19.1 vs. 10.2%, 10.5 and 8.3% for New South Wales, Victoria, and South Australia, respectively).

Results also showed differences for FDC schemes assessed under the 2012 and 2018 versions of the NQS. While similar proportions of schemes received a “WT” rating (47.8 and 47.0%, respectively), “EXCEED” ratings were less likely (7.6 vs. 19.5%), and “MEET” ratings were more likely (43.6 vs. 32.7%) for 2018 versus the 2012 version.

### Preliminary results

A multiple regression model may be invalid due to the existence of categorical variables in the study. A number of preliminary analyses were conducted to confirm the viability of a multiple regression analysis.

Possible differences in quality ratings based on the NQS version used were tested for. A preliminary MLR was conducted with the 2012 vs. 2018 version of the NQS as the IV and the overall NQS rating as the dependent variable. The model was statistically significant, χ^2^(2) = 15.416, *p* < 0.001. The version of the NQS was subsequently entered as a covariate in the MLRs for each of the four macro-structural characteristics. Next, we tested for model validity. For an MLR model to be valid: (1) there should be few covariate patterns with expected cell frequencies of zero; and (2) the proportion of expected cell frequencies greater than 5 should be 80% or more (34). Our preliminary analysis using the 4-category DV revealed multiple covariate patterns with expected frequencies of zero. In particular, we found that including the SIR rating led to covariate patterns with expected cell frequencies of zero. To avoid this problem, we combined the four FDC schemes with a “SIR” rating with schemes rated as “WT.” The combined “WT”/“SIR” category, therefore, consists of all FDC schemes that failed to meet at least one NQS element.

Our preliminary analyses also showed that entering more than one macro-structural predictor (IV) in the regression model resulted in invalid models. To create more trustworthy models, we reduced the number of categories for level of urbanization and jurisdiction. The three levels of urbanization with the highest remoteness levels – outer regional Australia (*n* = 47), remote Australia (*n* = 4), and very remote Australia (*n* = 2), were combined into a single category. For jurisdiction, the five states and territories with the lowest numbers of FDC schemes – Western Australia (*n* = 30), South Australia (*n* = 12), Tasmania (*n* = 10), ACT (*n* = 6), and NT (*n* = 3), were combined into a single sub-category. When the MLR was repeated with the new sub-categories, the models were valid. MLR was thus an appropriate method of analysis.

### Multinomial logistic regression

Results for the MLR models for each of the four macro-structural characteristics - management type, community SES, level of urbanization, and jurisdiction, all achieved statistical significance, explaining 19.3, 9.2, 6.9, and 7.8% (Nagelkerke *R*^2^) of the variance in overall NQS ratings, respectively (see Table 2).

**Table 2:** Results of multinomial logistic regression tests for each macro-structural characteristic.

Macro-structural characteristics (IV)		WT/SIR vs. EXCEED	WT/SIR vs. EXCEED	MEET vs. EXCEED
		Model *R*^2^ and	Model *R*^2^ and	Model *R*^2^ and
		Odds Ratios	Odds Ratios	Odds Ratios
Management type				
		*R*^2^ = 0.193	*R*^2^ = 0.193	*R*^2^ = 0.193
*Reference cat Private for-profit*	Not-for-profit community based	2.96***	8.86***	2.99*
Not-for-profit other Organization		3.35**	10.10***	3.01
				
Not-for-profit local government		2.74**	13.89***	2.23***
SES				
		*R*^2^ = 0.092	*R*^2^ = 0.092	*R*^2^ = 0.092
*Reference cat* *Quintile 3*	Quintile 1	0.44*	1.82	4.12
	Quintile 2	0.45	1.63	3.62
	Quintile 4	0.53	4.12	7.86**
	Quintile 5	0.57	4.50	7.88**
Urbanization level				
		*R*^2^ = 0.069	*R*^2^ = 0.069	*R*^2^ = 0.069
*Reference cat Major cities*	Inner regional Australia	1.94*	2.30*	1.19
	Outer regional + remote + very remote Australia	2.05	1.54	0.76
State/Territory Jurisdiction		*R*^2^ = 0.078	*R*^2^ = 0.078	*R*^2^ = 0.078
*Reference cat* *Queensland*	New South Wales	0.63	0.46	0.73
	Victoria	0.53	0.33**	0.62
	Western Australia + South	0.32*	0.50	1.56
	Australia + Tasmania +			
	ACT + Northern Territory			

* *p* < 0.025,

** *p* < 0.01,

*** *p* < 0.001.

Management type had a moderate effect on FDC quality,χ^2^(8) = 80.31, *p* < 0.001, Pearson’s χ^2^(6) = 8.89, *p* = 0.180, *R*^2^ = 0.193. Significant Odds Ratios were evident for all three MLR comparisons. These are summarized using for-profit FDC as the reference category in Table 2. Results showed that for-profit schemes were more likely to be rated “WT”/“SIR” compared to “MEET” than community-based not-for-profit FDC schemes (OR = 2.96; *p* < 0.001), FDC schemes managed by other not-for-profit organizations (OR = 3.35; *p* = 0.002), and local government managed FDC (OR = 2.74; *p* = 0.001). For-profit FDC schemes were also more likely to be rated “WT”/“SIR” compared to “EXCEED” than community-based not-for-profit FDC schemes (OR = 8.86; *p* < 0.001), FDC schemes managed by other not-for-profit organizations (OR = 10.01; *p* < 0.001), and local government FDC schemes (OR = 13.88; *p* < 0.001). For- profit FDC schemes were more likely to be rated “MEET” compared to “EXCEED” than community-based not-for-profit FDC schemes (OR = 2.99; *p* = 0.017), and local government FDC schemes (OR = 5.06; *p* < 0.001).

Community SES had a small but significant effect on FDC quality ratings, χ^2^(10) = 34.29, *p* < 0.001; Pearson’s χ^2^(8) = 6.74, *p* = 0.565, *R*^2^ = 0.092. All five levels of SES (SEIFA quintiles 1 to 5) were tested as the reference category in a series of MLR tests to explore the overall effect. Results are summarized for Odds Ratio comparisons for quintile 3, the mid-point, in Table 2. Significant Odds Ratios were evident for two MLR comparisons. FDC schemes in quintile 3 were less likely to be classified as “WT”/“SIR” compared to “MEET” than FDC schemes in quintile 1 (OR = 0.44; *p* = 0.011) and more likely to be classified as “MEET” compared to “EXCEED’ than FDC schemes in quintile 4 (OR = 7.86; *p* = 0.004), and SEIFA quintile 5 (OR = 7.88; *p* = 0.007).

Level of urbanization had a small effect on FDC quality ratings, χ^2^(6) = 27.19, *p* < 0.001, Pearson’s χ^2^(4) =7.70, *p* = 0.103, *R*^2^ = 0.069. Results are summarized using major cities of Australia as the reference category. FDC schemes in major cities of Australia were significantly more likely to be (1) rated “WT”/“SIR” compared to “MEET” and (2) rated “WT”/“SIR” compared to “EXCEED” than schemes in inner regional Australia (ORs = 1.94; *p* = 0.014, and 2.30; *p* = 0.022), respectively.

Government jurisdiction showed a small but significant difference in FDC quality ratings [χ^2^(8) = 30.66, *p* < 0.001; Pearson’s χ^2^(6) =8.66, *p* = 0.193, *R*^2^ = 0.078] based on our four-level categorization that compared New South Wales, Victoria, Queensland and combined group of five jurisdictions. While all categories were tested as the reference category in a series of MLR tests, Odds Ratios results are summarized for Queensland as the comparison state. FDC schemes managed by Queensland were less likely to be rated “WT”/“SIR” compared to “MEET” than FDC schemes managed by the combined group (OR = 0.32; *p* = 0.003), and less likely to be rated “WT”/“SIR” compared to “EXCEED” than FDC schemes managed by Victoria (OR = 0.33; *p* = 0.007). In addition, MLR results using Victoria as the reference category showed that FDC schemes managed by Victoria were more likely to be rated “WT”/“SIR” compared to “MEET” than the combined FDC schemes managed by the combined group (OR = 3.13; *p* = 0.003). MLR results for New South Wales as the reference category indicated no differences in quality ratings for jurisdiction.

## Discussion

The study results provide clear evidence that macro-structural characteristics predicted differences in FDC quality. This section will discuss these findings and their implications for process quality.

### Management type

For-profit FDC schemes had lower quality ratings than all three types of not-for- profit FDC schemes. The magnitude of the difference between for-profit and not-for-profit FDC services analyzed in this study was surprising. 60% of for-profit FDC schemes failed to meet the National Quality Standard, over twice the rate for not-for-profit schemes. This gap between for-profit and not-for-profit FDCs is of particular concern, as for-profit FDC schemes make up over 56% of all FDC schemes in Australia.

Market forces has been suggested to explain the difference in quality by management type. The thick and thin market theory of childcare quality posits that middle- to upper-income families may influence quality through market forces, with better child-to-staff ratios and higher fees (19). However, this theory holds key assumptions that differ from the Australian FDC context. Australia’s National Quality Framework ensures that child-to-staff ratios are consistent across FDCs. Furthermore, government subsidies are available to parents for all FDCs regardless of management type.

Differences in quality has been suggested to arise from differences in educators’ rates of pay. For-profit providers of center-based services pay educators lower rates and are less likely to attract highly-qualified and well- experienced staff than not- for-profit providers. They also pay high salaries for executives and dividends to shareholders (38). Applying these findings to FDC may explain the for-profit/not-for-profit quality gap. For-profit FDC schemes may pay coordinators and fieldworkers lower wages than not-for-profit providers and direct profits to the executives, shareholders, and/or owners rather than to the resourcing and support of FDC educators.

### Community socioeconomic status

A general pattern of FDC schemes located in lower SES communities having lower NQS ratings and FDC schemes in communities of higher SES having higher ratings was found. The findings were similar to that of other research [e.g., (5, 21)]. This pattern could be explained by the theory of concentrated affluence and concentrated disadvantage (39). Areas of concentrated affluence are defined by having many families earning more than $100,000 annually and having a member with at least a Bachelor’s degree. On the other hand, areas of concentrated disadvantage have high levels of unemployment and a high proportion of families living in poverty (40). FDC educators residing in communities with higher concentrated affluence may be more likely to have tertiary qualifications and be more invested in providing higher-quality programs. Likewise, families with tertiary qualifications and higher incomes may purposely seek higher-quality FDC homes and thus create demand for them.

### Level of urbanization

The present study found that FDC schemes in regional areas of Australia attained higher levels of quality than services in metropolitan areas. This finding contrasts with previous studies reporting higher quality ECEC in metropolitan areas for kindergartens and LDC centers (4, 25, 41). The difference for FDC quality may be explained, in part, by the higher level of accessibility to FDC in regional and remote Australia: 28% of the Australian population live in regional and remote areas of Australia (42), and 24.3% of FDC educators operate in the same regions (3). In addition, FDC may be the only option for child care in regional and remote areas of Australia (3). In contrast, households in metropolitan Australia are more likely to experience multiple challenges accessing child care (e.g., lack of quality, center location, and center choice) than households in regional Australia or remote Australia (43).

### State/territory government jurisdiction

Finally, our findings also identified jurisdiction as a predictor of FDC quality. FDC schemes in Queensland were generally found to be of higher quality than FDC schemes other jurisdictions, with the exception of NSW. Since 2012, all states and territories have been expected to adhere to national guidelines for quality; however, before the reforms introduced in 2012, there were disparities in standards and administrative processes for the child-to-staff ratios and educators’ qualifications. It may be that these prior distinctions continue to affect current levels of quality.

### Process quality

Overlap between the NQS Quality Areas (QAs) and standardized assessments of quality has been reported by Siraj et al. (44) who found associations between QA 1 (education program and practice) and QA 5 (relationships with children) and independent ratings on the Sustained Shared Thinking and Emotional Wellbeing (SSTEW) scale. QA 1 assesses educators against their use of child-centered opportunities, intentional teaching, responsive teaching, and child directed learning: areas that emphasize curriculum quality. QA 5 assesses educators against their interactions with children, promotion of collaborative learning, and promotion of child self-regulation (4). Given that MLR tests explained between 6.9 and 19.3% of variation in NQS overall ratings, the findings from this study suggest that (1) curriculum quality; and (2) relationships with children are significantly associated with macro-structural characteristics of the FDC services. If so, these findings suggest that improvements in process quality may be best served through a holistic approach emphasizing direct interventions at the FDC service level in combination with indirect macro-structural interventions.

### Limitations

While our use of a publicly available, large, national data set to examine macro-structural differences in FDC quality has made a unique contribution, these data have inherent limitations. FDC assessment and rating is conducted in a sample of FDC homes that are expected to represent the large number of individual FDC homes in each scheme. A further limitation is that, unlike in the U.S., the assessment process does not use of standardized observation instruments and, while accepted as conceptually sound (45, 46), only one validation of the NQS ratings has been conducted (44). To our knowledge, no validation study of FDC ratings has been undertaken in Australia. A further limitation is that FDC schemes include educators over wide geographical areas (47). The SEIFA score assigned to FDC schemes may not necessarily match the SEIFA of individual FDC educators.

Second, the small numbers of schemes in Western Australia, South Australia, Tasmania, the ACT, and Northern Territory were combined into one group to ensure valid regression models, but this was not a coherent group. Our initial analyses (summarized in Table 1) showed differences in the distribution of FDC quality within these five states/territories. Conducting MLRs with categorical outcomes and predictors also limited our ability to test and compare the combined effects of the four macro-structural variables and covariates simultaneously in a multivariable regression.

## Conclusion

Given that FDC is a widely utilized yet seldomly researched form of ECEC, the potential offered by Australia’s NQS dataset has realized important findings. The Australian NQS is unique in applying a universal quality improvement and rating system across all jurisdictions. It is also tied to standardized regulations for child-educator ratios, educator qualifications, and the Australian government’s childcare fee subsidy system. The NQS is a relatively novel measure of quality in FDC, about which relatively little is known from a research perspective. This study contributes to ongoing research about macro-structural characteristics that predict FDC quality and research about predictors of NQS quality indicators. Findings imply that policy attention should be paid to the variations and inequalities linked to macro-structural characteristics to promote equality and equity in FDC services.

## Data availability statement

Publicly available datasets were analyzed in this study. This data can be found at: acecqa.gov.au.

## Author contributions

This article reports VC’s postgraduate study, co-supervised by LH and HL, at Macquarie School of Education, Macquarie University. All authors contributed to the article and approved the submitted version.

## Conflict of interest

The authors declare that the research was conducted in the absence of any commercial or financial relationships that could be construed as a potential conflict of interest.

## Publisher’s note

All claims expressed in this article are solely those of the authors and do not necessarily represent those of their affiliated organizations, or those of the publisher, the editors and the reviewers. Any product that may be evaluated in this article, or claim that may be made by its manufacturer, is not guaranteed or endorsed by the publisher.
